# Balloon pulmonary angioplasty combined with riociguat for the treatment of inoperable chronic thromboembolic pulmonary hypertension (PRACTICE study): study protocol for a randomized controlled trial

**DOI:** 10.1186/s13063-021-05910-5

**Published:** 2021-12-27

**Authors:** Qin-Hua Zhao, Su-Gang Gong, Jing He, Ping Yuan, Wen-Hui Wu, Ci-Jun Luo, Rong Jiang, Rui Zhang, Hong-Ling Qiu, Hui-Ting Li, Yuan Li, Jin-Ming Liu, Lan Wang

**Affiliations:** Department of Pulmonary Circulation, Shanghai Pulmonary Hospital, Tongji University School of Medicine, Zhengmin Road, Shanghai, 200433 China

**Keywords:** Randomized controlled trial, Balloon pulmonary Angioplasty, Riociguat, Chronic thromboembolic Pulmonary hypertension

## Abstract

**Background:**

Management of inoperable chronic thromboembolic pulmonary hypertension (CTEPH) remains a clinical challenge. Currently, riociguat, a soluble guanylate-cyclase stimulator is recommended by international guidelines. More recently, balloon pulmonary angioplasty (BPA) develops as an alternative treatment for inoperable CTEPH.

**Method:**

This study is a single-center randomized controlled trial. Subjects with inoperable CTEPH are randomized into either a BPA combined with riociguat or riociguat monotherapy group (2:1) and observed for 12 months after initiation of treatment. The primary endpoint is the change in pulmonary vascular resistance from baseline to 12 months after initiation of treatment. The secondary endpoints include 6-min walk distance (6MWD), WHO-FC, NT-proBNP, SF-36, and other hemodynamic parameters. Safety endpoints are analyzed too.

**Discussion:**

This study aims to compare the efficacy and safety of BPA combined with riociguat and riociguat monotherapy for inoperable CTEPH.

**Trial registration:**

Chinese Clinical Trial Registry ChiCTR2000032403. Registered on 27 April 2020.

## Background

Chronic thromboembolic pulmonary hypertension (CTEPH) is a complex chronic disease. Pulmonary artery stenosis or obstruction caused by organized thrombus can lead to increased pulmonary artery pressure and pulmonary vascular resistance, ultimately triggering progressive right heart failure and death [1]. As a serious long-term complication of acute pulmonary embolism, its exact mechanism is not fully understood and the prognosis is extremely poor. The 5-year survival rate of untreated CTEPH patients with a mean pulmonary artery pressure > 40 mmHg is 30%, while it is only 10% in CTEPH patients with a mean pulmonary artery pressure > 50 mmHg [2]. Currently, the treatment of the CTEPH is limited, mainly including pulmonary endarterectomy (PEA), target treatment medications, and balloon pulmonary angioplasty (BPA) [3–5].

PEA is an established curative treatment and is recommended for patients with operable CTEPH as the first choice by the European Society of Cardiology/European Respiratory Society (ESC/ERS), Cologne Consensus, and Chinese Pulmonary hypertension diagnosis and treatment guidelines (I, C) [3–5]. Nevertheless, 20-40% of patients are deemed unsuitable for PEA surgery due to comorbidities and other factors, and nearly half of the operated patients have residual or recurrent pulmonary hypertension [6–8].

Riociguat, a soluble guanylate-cyclase (sGC) stimulator, has a direct stimulating effect on sGC, which is independent of nitric oxide (NO) level. Meanwhile, it also increases the sensitivity of sGC to NO [9]. These two effects increase the level of cyclic guanosine monophosphate (cGMP), which induces vasodilation and suppresses vascular remodeling, inflammation, and platelet aggregation [10]. Riociguat gets FDA approval in inoperable or persistent/recurrent CTEPH based on the findings of a multicenter randomized clinical trial (CHEST-1) [11] and its extension study (CHEST-2) [12].

BPA is a catheter-based treatment, which is also reported to be effective for inoperable CTEPH [13–17]. After more than 30 years of development and refinements, emerging evidence confirms its role in patients with inoperable CTEPH or residual/recurrent pulmonary hypertension with acceptable complications and comparable long-term prognosis to PEA. Therefore, BPA is a promising alternative treatment for patients with CTEPH and is recommended by the current guidelines for the treatment of inoperable PEA surgery or persistent/recurrent CTEPH after PEA (IIb) [3–5]. However, the modified BPA technology also has certain limitations. It cannot expand distal diffuse pulmonary vascular disease and cannot stop pulmonary vascular remodeling; meanwhile, medications have a very strong effect on mechanical obstruction caused by micro-thrombosis, suggesting that medications and BPA have a different mechanism on the treatment of CTEPH, and the combination of the two methods may be better to further improve CTEPH patients’ outcomes.

The aim of this study is to evaluate the efficacy and safety of BPA combined with riociguat for inoperable CTEPH over 12 months. The results of this study may aid in optimizing treatment selection and improving patient outcomes.

### Hypotheses

The primary hypothesis for the trial is:

1. For PEA-inoperable patients, BPA combined with riociguat significantly improves hemodynamic parameters including pulmonary vascular resistance compared with riociguat alone.

The secondary hypotheses are:

1. BPA combined with riociguat significantly improves activity tolerance including 6-min walk distance, WHO functional class, SF-36 compared with riociguat alone.
2. BPA combined with riociguat does not have serious adverse events compared with riociguat alone. It has good safety and tolerance.

## Methods and analysis

### Design

**Fig. 1:**
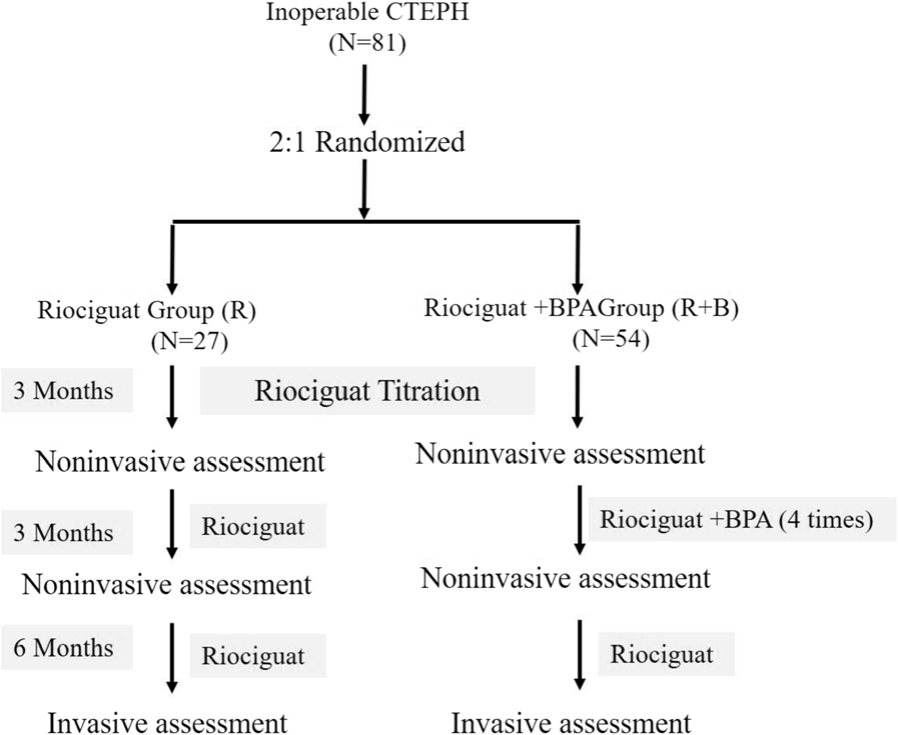
Flow diagram of study recruitment and randomization. After obtaining consent and diagnosis of chronic thromboembolic pulmonary hypertension is confirmed, subjects will be randomized into either the B+R or R group, to receive treatment for 12 months. Patients in the B+R group receive 4 times BPA 3 months later initial riociguat in 3 months. Observations will be recorded at the time of screening/baseline and at 3, 6, and 12 months after the initiation of treatment. BPA, balloon pulmonary angioplasty; CTEPH, chronic thromboembolic pulmonary hypertension

This study is a singer-center, prospective, randomized controlled trial. As shown in Fig. 1, subjects willing to consider enrollment are consented prior to invasive evaluation. Subjects undergo V/Q scan, right-heart catheterization, and pulmonary angiography for definitive diagnosis of CTEPH by the standard practice based on ESC guideline recommendation [3]. The identified CTEPH patients are provisionally enrolled in this study. An independent experienced PEA surgeon determines if subjects are eligible for PEA. If the subjects are deemed technically operable, they are excluded from this study. All patients who meet the inclusion criteria but don’t meet the exclusion criteria are randomly divided into two groups with 2:1 rate via a randomization application and are observed for 12 months. One group is treated with riociguat only, namely the riociguat group (R group); the other group is given additional BPA therapy on the basis of 3 months riociguat treatment, which is riociguat + BPA group (R+B group). Riociguat tablets are administered orally to all randomized patients at starting dose of 1.0 mg TID and are increased by 0.5 mg increments in 2-week intervals to 1.5, 2.0, and 2.5 mg TID (maximum total daily dose: 7.5 mg) if systolic blood pressure (SBP) is ≥ 95 mmHg and there are no signs or symptoms of hypotension. If higher doses are not tolerated, patients are maintained on lower doses (minimum allowed dose: 0.5 mg TID). After the end of the 8-week titration phase, riociguat is continued at the optimal individual dose for 4 weeks. In the R+B group, patients receive 4 times BPA in 2–3-week intervals in the following 3 months according to the morphology of the pulmonary lesion evaluated by preoperative right-heart catheterization and pulmonary angiography. In general, 3 vessels are performed each time. However, at least among the collaborative institutions included in this study. PEA-inoperable patients are very rarely considered unsuitable for BPA, because the institutes are professional BPA centers in China. Then all participants are followed up to 12 months. And the safety follow-up is up to 30 days later the study completion.

Observations are made at the time of screening or baseline and 3, 6, and 12 months after initial treatment. All BPA procedures are performed by two specific surgeons who are blinded to the trial, and echocardiography, 6MWD, and cardiopulmonary exercise testing are performed by specific experienced physicians who are blinded to the trial.

Data processing of patients who withdraw from the study or terminate the study uses the last measured value (LOCF) before withdrawal for statistical analysis. Table 1 shows the schedule of assessments performed at each visit for each treatment group, including mandatory and optional assessments.

**Table 1:** Schedule of visits and observation items for enrolled subjects

Observation items	Visit 1 Screening/baseline	Visit 2 3 months ± 7 days	Visit 3 6 months ± 7 days	Visit 4 12 months ± 7 days
Informed consent	X			
Inclusion/exclusion	X			
Demographics	X	X	X	X
Medical history	X	X	X	X
vital signs	X	X	X	X
Laboratory tests	X	X	X	X
Blood gas test	X	X	X	X
electrocardiogram	X	X	X	X
6MWD	X	X	X	X
WHO-FC	X	X	X	X
Pulmonary function	X			X
Echocardiography	X		X	X
RHC	X			X
CPET	X		X	X
CMR	X		X	X
QOL (SF-36)	X	X	X	X
Concomitant medication	X	X	X	X
Medication adherence		X	X	X
Adverse events	X	X	X	X

*6MWD* 6-min walk distance, *WHO-FC* World Health Organization functional class, *RHC* right-heart catheterization, *CPET* cardiopulmonary exercise testing, *CMR* cardiac magnetic resonance, *QOL* quality of life

### Ethics

The trial is approved by the local ethics committee (EC) of Shanghai Pulmonary Hospital (L20-385-1). The whole procedures for trial implementation, evaluation, and filing stated in this plan are designed to ensure that researchers follow the guidelines of the ‘Clinical Trial Management Practice’ and the ‘Declaration of Helsinki’. The investigator can change the plan to eliminate the possible risks that the patient may face without the EC approval. Explains and amendments should be immediately submitted to the EC. Written informed consent should be obtained from all participants after a full explanation of this study (Trial registration number: ChiCTR2000032403).

### Sample size

Previously, BPA shows to result in reducing pulmonary vascular resistance from 7.3±3.2 WU to 3.8±1.0 WU (mean ± SD) within 6 months [15], while riociguat is reported to decrease pulmonary vascular resistance by 2.83±3.1 WU [6]. Based on these studies, it is assumed that the change in mean pulmonary vascular resistance from the start of treatment to 12 months after treatment would be − 4.5 WU for the BPA+R group and − 2.8 WU for the R group, with SD of 2 and 2, respectively. The minimum sample size requires to achieve a significance of 0.05 from a two-sided test with a statistical power of 90%. The combination group is determined to be 54 subjects, and the riociguat group is 27 subjects, for a total of 81 subjects. We estimate the dropout rate to be 15%.

### Eligibility criteria

Inclusion criteria include the following: (a) diagnosis with CTEPH with a WHO functional class II or III based on the diagnostic criteria of the 2015 ESC/ERS guidelines [3]; (b) age ≥ 18 and < 75 years; (c) mean pulmonary arterial pressure of ≥25 mmHg, pulmonary artery wedge pressure of ≤15 mmHg and pulmonary vascular resistance of ≥4.5 WU; (d) administration of appropriate anticoagulant therapy for at least 3 months; (e) provision of written informed consent; (f) patients with non-central CTEPH are not suitable for PEA surgery, or the patient refuses to undergo PEA surgery, or the patient’s general condition is poor and cannot tolerate PEA surgery.

Exclusion criteria include the following: (a) participate in other clinical trials within 3 M before visit 1 or participate in other clinical trials; (b) group 1, 2, 3, 5 pulmonary hypertension; (c) refuse to sign the informed consent form, or unable to cooperate with the investigator during the trial; (d) refuse or unsuitable to BPA; (e) use other targeted medications and other drugs that cannot be combined with riociguat; (f) pregnancy or breastfeeding; (g) contraindicated for riociguat; (h) accompanied by severe chronic or acute liver, kidney or central nervous system diseases; (i) life expectancy of less than 1 years; (j) accompanied by unstable angina, or have a history of myocardial infarction, stroke or life-threatening arrhythmia within 6 months before this test; (k) cannot supine for a long time due to mental factors; (l) patients in the acute infection period; (m) active hemoptysis; (n) obvious obstructive or restrictive lung disease; (o) deemed to be unsuitable for participation by the investigators.

Withdrawal criteria include the following: (a) patient or his legal representative requests to withdraw from the trial; (b) the investigator believes that continuing the experiment will damage the patient's health; (c) in any case, the reason for withdrawal must be recorded in the case report form and the patient's medical record; (d) all patients who withdraw from the trial due to adverse events or abnormal clinical laboratory tests should be followed up until they recover or their condition is stable, and the results should be recorded.

### Recruitment and consent

The informed consent document is presented to potentially eligible subjects to provide a comprehensive explanation of this study. Subjects willing to consider enrollment are consented prior to right-heart catheterization and pulmonary angiography for definitive diagnosis of CTEPH. However, to reduce the burden on the subjects, suitable patients that had undergone comprehensive evaluation, including right-heart catheterization and pulmonary angiography, within 3 months before the consenting can also be enrolled in this study. This is explained to subjects by investigators at the time of obtaining consent. Once consent is obtained, an independent experienced PEA surgeon who is not involved in this study determined operability (whether subjects are eligible for PEA) based on imaging data according to the Guidelines for Treatment of Pulmonary Hypertension [3]. Random assignment is performed centrally and research institutions by biased-coin minimization. In this study, a third-party statistician uses SAS software to perform randomization. The patients are divided randomly through random numbers obtained by the software.

### Outcome measure

The primary endpoint is the change in pulmonary vascular resistance between baseline and 12 months. Secondary endpoints include several clinical and quality-of-life parameters (as detailed in Table 2).

**Table 2:** Secondary endpoints, exploratory endpoint and safety evaluation that will be measured and/or compared at baseline and at 12 months after initiation of treatment

Secondary endpoints	
Change in hemodynamic variables	mean pulmonary arterial pressure, cardiac output, etc.
Change in 6-min walk distance	
Change in WHO functional class	
Change in plasma NT-proBNP level	
Change in echocardiography	tricuspid annulus systolic displacement (TAPSE), right atrium area, right ventricle size, eccentricity index, pericardial effusion
Change in Borg dyspnea index	
Change in quality-of-life parameters (SF 36)	
**Exploratory endpoint**	
cardiopulmonary exercise testing	
cardiac magnetic resonance	
**Safety evaluation**	
Frequency of adverse events	hemoptysis/pulmonary hemorrhage (vascular perforation, vascular dissection, vascular rupture, etc), pneumothorax, hypotension, pulmonary congestion/ pulmonary edema, late-onset lung disturbance, heart failure, pneumonia, headache, dizziness, peripheral edema, nausea/vomiting, retching, diarrhea, nasopharyngitis, upper respiratory inflammation, respiratory distress, coughing and fainting
Clinical worsening during the observation period and time to clinical worsening	All-cause mortality, heart/lung transplant, hospitalization due to clinical worsening, new initiation of pulmonary hypertension target medications, worsening of 30% or greater from baseline in the 6MWD and persistent worsening in the WHO FC from baseline due to the worsening of a primary disease.
Laboratory tests	hemoglobin, alanine aminotransferase, creatinine, aspartate aminotransferase

### Data collection and management

The investigators who enter information into the case report form (CRF) are responsible for ensuring the accuracy and completeness of information. The investigators must save the CRF and all the original data. According to CRF data, a database is established through Epidata software. Data collection and management are carried out by physicians who are blinded to this trial to avoid bias. Linkable anonymization by central registration number is used to identify the subjects and only the subject number is recorded in the case report form. Moreover, when a patient’s name appears in any other document (such as a pathology report), the name must be hidden before providing a copy of the document. The investigators are responsible for the appropriate storage of the correspondence table prepared by them to identify the subjects. This correspondence table must be retained for 5 years after completion of this study. Appropriate measures, such as encoding or deletion, are taken to ensure that the subjects cannot be identified in any display or public disclosure of information related to this study, in accordance with applicable laws and regulations. If data are transmitted over an unsecured electronic network, the data must be encoded at the source. Representatives of the clinical research office, ethics committee/institutional review committee, and relevant supervision and management departments have the right to inspect medical records to verify the collected data. During the inspection process, the above-mentioned personnel strictly follow the local data protection laws to keep the subjects’ personal information confidential.

If the results of the study are published publicly, the information confirming the identity of the subject remains confidential.

### Frequency and plans for auditing trial conduct

Auditors will verify adherence to required clinical trial procedures and will confirm accurate data collection according to the Good Clinical Practice (GCP) guidelines. Study monitoring and follow-up, from the initial setup to final reporting, will be fulfilled according to current National and International requirements.

### Provisions for post-trial care

Before study entry, potential participants are informed via the consent form that they will be offered post-trial treatment according to routine clinical practice.

### Adverse events

The occurrence of any untoward medical events, including complications or the worsening of pre-existing underlying diseases, is defined as adverse events. Worsening of efficacy evaluation indices is not defined as adverse events. Any concomitant symptoms or clinically significant abnormal fluctuations in test results are investigated to determine whether there is a cause-and-effect relationship with BPA or riociguat, and findings are documented in the CRF. Adverse events are followed up until normalization or recovery to a level not considered to be an adverse event, or in the case of an irreversible adverse event (cerebral infarction, myocardial infarction, etc.), until symptoms stabilize.

### Statistical analysis

Data analysis is performed by a specific specialized investigator who keeps being blind to the trial. The research measurement data are presented as mean ± standard deviation, and count data are presented as frequency or percentage. In the baseline characteristics of patients, count data are compared using Pearson’s *χ*^2^ test or Fisher’s exact test and measurement data are compared using *t* test. For primary analysis, the least square means difference and 95% CI for change of pulmonary vessel resistance between groups at 12 months are estimated using analysis of covariance adjusted for allocation factors. For patients who withdraw from the study, the last observation carried forward (LOCF) before withdrawal is used for statistical analysis. Adverse events are evaluated during the safety analysis. The frequencies of adverse events are compared using Fisher’s exact test. We consider the *p* value < 0.05 of two-sided test to be statistically significant. All statistical analysis is performed using SPSS software V22.0. The statistical analysis plan is developed by the principal investigator and biostatistician before the completion of patient recruitment and data fixation.

## Discussion

2015 ESC/ERS Guidelines for the diagnosis and treatment for pulmonary hypertension, 2018 Cologne Consensus, and 2018 Chinese pulmonary hypertension diagnosis and treatment guidelines recommend riociguat and BPA for patients with inoperable CTEPH [3–5]. Sequential treatment with riociguat and BPA for patients with inoperable CTEPH shows significant improvements in mean pulmonary arterial pressure and pulmonary vascular resistance [18]. Case report shows hybrid of riociguat and BPA therapy in CTEPH patient improved echocardiographic parameters of RV function [19]. A randomized controlled trial comparing riociguat and BPA (the RACE study) is being conducted in France (https://clinicaltrials.gov/ct2/show/NCT02634203). A multicenter randomized controlled trial to compare BPA versus riociguat in patients with CTEPH is ongoing [20]. However, there is no report directly comparing treatment outcomes of BPA combined riociguat and riociguat alone. Therefore, this study is planned to compare the efficacy and safety of BPA combined with riociguat and riociguat alone for inoperable CTEPH.

According to the current guidelines, the diagnosis of CTEPH requires at least 3 months of anticoagulation. Therefore, 3 months is set as the anticoagulation course for newly diagnosed patients with suspected CTEPH to exclude patients with subacute thrombosis. For CTEPH patients who have been previously diagnosed and have been anticoagulated for a long time, they can be directly evaluated. The primary endpoint of this study is the change in pulmonary vascular resistance from baseline to 12 months, as this is an important factor to decide the severity of CTEPH. Other outcomes often used in studies on CTEPH, such as the 6MWD and mean pulmonary arterial pressure, are secondary endpoints in this study. In this study, 4 times BPA treatment is completed in 3 months and 3 vessels are performed each time, which is in keeping with real-world practice in China. This study also evaluate the long-term efficacy and safety of BPA combined with riociguat and riociguat alone by continuing evaluations for 12 months after initiation of treatment. In addition, the operability of PEA is determined by an independent experienced PEA surgeon who belongs to an independent institute. This avoids recruitment bias and guarantees reliability in this study. Furthermore, this study compares patient-reported quality-of-life and the parameters from CMR. These factors may contribute to optimizing treatment strategies and following-up strategies for inoperable CTEPH. The results of this study are disseminated at medical conferences and in journal publications.

### Strengths and limitations of this study

This is a randomized controlled trial comparing the efficacy and safety of BPA combined with riociguat and riociguat alone in patients with inoperable chronic thromboembolic pulmonary hypertension. This study evaluates the efficacy and safety of BPA combined with riociguat over a relatively long period (12 months). This is the first study to compare the efficacy and safety between BPA combined with riociguat and riociguat alone.

The main limitation of this study is the open-label trial design. Because there is no distinct criterion for BPA in each lesion, operators are aware of the mean pulmonary vascular resistance (the primary endpoint in this study), and they might be incentivized to continue BPA. Thus, the bias for the BPA operators cannot be completely avoided. However, since this study compares medical and surgical treatment, it is difficult to use a placebo or mask patients and/or physicians. Other limitations of this study include single-center design and a relatively small number of subjects are recruited.

## Data Availability

The main trial datasets generated and/or analyzed during the current study are not publicly available while the study is underway due to the sensitivity of the data, but are available from the corresponding author on reasonable request. Data is however available from the authors upon reasonable request and with permission of the Shanghai Pulmonary Hospital Database.
